# Sulindac targets nuclear *β*-catenin accumulation and Wnt signalling in adenomas of patients with familial adenomatous polyposis and in human colorectal cancer cell lines

**DOI:** 10.1038/sj.bjc.6601505

**Published:** 2004-01-06

**Authors:** E M J Boon, J J Keller, T A M Wormhoudt, F M Giardiello, G J A Offerhaus, R van der Neut, S T Pals

**Affiliations:** 1 Department of Pathology, Academic Medical Center, University of Amsterdam, Meibergdreef 9, Amsterdam 1105 AZ, The Netherlands; 2 Department of Medicine, Johns Hopkins Medical Institutions, Baltimore, MD 21205, USA

**Keywords:** sulindac, TCF, FAP

## Abstract

Nonsteroidal anti-inflammatory drugs (NSAIDs) have chemopreventive potential against colorectal carcinomas (CRCs). Inhibition of cyclooxygenase (COX)-2 underlies part of this effect, although COX-2-independent mechanisms may also exist. Nonsteroidal anti-inflammatory drugs appear to inhibit the initial stages of the adenoma–carcinoma sequence, suggesting a link to the APC/*β*-catenin/TCF pathway (Wnt-signalling pathway). Therefore, the effect of the NSAID sulindac on nuclear (nonphosphorylated) *β*-catenin and *β*-catenin/TCF-mediated transcription was investigated. Nuclear *β*-catenin expression was assessed in pretreatment colorectal adenomas and in adenomas after treatment with sulindac from five patients with familial adenomatous polyposis (FAP). Also, the effect of sulindac sulphide on *β*-catenin/TCF-mediated transcription was studied. Adenomas of FAP patients collected after treatment with sulindac for up to 6 months showed less nuclear *β*-catenin expression compared to pretreatment adenomas of the same patients. Sulindac sulphide abrogated *β*-catenin/TCF-mediated transcription in the CRC cell lines DLD1 and SW480, and decreased the levels of nonphosphorylated *β*-catenin. As a result, the protein levels of the positively regulated TCF targets Met and cyclin D1 were downregulated after sulindac treatment. This study provides *in vivo* and *in vitro* evidence that nuclear *β*-catenin localisation and *β*-catenin/TCF-regulated transcription of target genes can be inhibited by sulindac. The inhibition of Wnt-signalling provides an explanation for the COX-2-independent mechanism of chemoprevention by NSAIDs.

Epidemiological data, rodent studies and *in vitro* experiments have demonstrated that nonsteroidal anti-inflammatory drugs (NSAIDs) have anticolorectal cancer (CRC) activity (Giardiello *et al*, 1995). Also, in patients with familial adenomatous polyposis (FAP), an autosomal dominantly inherited disorder characterised by the development of numerous colorectal adenomas at a young age, the NSAIDs sulindac and indomethacin can cause regression of adenomas (Giardiello *et al*, 1993; Nugent *et al*, 1993; Spagnesi *et al*, 1994; Hirota *et al*, 1996; Winde *et al*, 1997; Picariello *et al*, 1998). The chemopreventive effect of NSAIDs appears mediated by the induction of apoptosis and cell cycle arrest (Pasricha *et al*, 1995; DuBois and Smalley, 1996; Piazza *et al*, 1997; Keller *et al*, 1999; Shiff and Rigas, 1999). The molecular mechanisms underlying these biological effects are not completely understood. Nonsteroidal anti-inflammatory drugs inhibit the enzymatic activity of cyclooxygenase (COX)-1 and -2, enzymes that convert arachidonic acid into prostaglandins (Shiff and Rigas, 1999). However, COX-independent mechanisms may also play a role, since NSAIDs inhibit the growth of colon cancer cell lines lacking COX-2 expression (Hanif *et al*, 1996; Zhang *et al*, 1999; Smith *et al*, 2000).

Oncogenic activation of the Wnt-signalling pathway by mutations in Adenomatous polyposis coli (APC) or *β*-catenin, which results in the accumulation and nuclear translocation of *β*-catenin and in *β*-catenin/TCF4-regulated transcription of TCF target genes, is mandatory for the initial neoplastic transformation of intestinal epithelium (reviewed in Kinzler and Vogelstein, 1996; Bienz and Clevers, 2000). Previous studies have shown an effect of NSAIDs on the expression and localisation of *β*-catenin, suggesting nuclear *β*-catenin as an alternative target for the chemopreventive effect of NSAIDs. Nonsteroidal anti-inflammatory drugs were shown to prevent the nuclear accumulation of *β*-catenin in chemically induced colon tumours in rats (Brown *et al*, 2001) and in human colorectal cancer cell lines (Smith *et al*, 2000; Hawcroft *et al*, 2002). In addition, indomethacin and aspirin can downregulate the expression of the TCF target gene cyclin D1 in CRC cell lines (Dihlmann *et al*, 2001; Hawcroft *et al*, 2002). Together, these data suggest that NSAIDs may exert an antineoplastic effect by inhibiting the Wnt-signalling pathway. Previously, we reported low levels of nuclear *β*-catenin in sulindac-resistant adenomas (Keller *et al*, 2001). This could reflect a downregulation of nuclear *β*-catenin by sulindac or represent an intrinsic feature of resistant adenomas. In the present study, we therefore compared nuclear accumulation of *β*-catenin in adenomas from FAP patients before and after treatment with sulindac for up to 6 months (Giardiello *et al*, 1993). In addition, we studied the effects of the active metabolite of sulindac, sulindac sulphide, on Wnt-signalling in human CRC cell lines.

## MATERIALS AND METHODS

### Patients and adenoma specimens

The study population consisted of five FAP patients who were treated with sulindac 150 mg p.o. twice a day as described previously (Giardiello *et al*, 1993, 1996; Cruz-Correa *et al*, 2002). All patients had adenomas at the initiation of treatment (baseline) and showed adenoma regression after 6 months treatment with sulindac. Patients were selected on the basis of availability of both adenomas collected at baseline and during the first 6 months of treatment with sulindac. Patient characteristics are shown in Table 1

**Table 1:** Patient characteristics

					**Polyp count**		**Polyps studied**	
**Patients**	**Age (years)**	**Sex**	**APC mutation**	**Colectomy/intact colon**	**Baseline**	**6 months**	**Baseline**	**After sulindac**
I	32	F	Not tested	IRA	19	3^a^	2	1
II	42	F	Codon 423	IRA	47	6^a^	2	6
III	52	F	Not tested	IRA	10	0	3	2^b^
IV	25	F	Codon 1061	IRA	7	0	1	2^b^
V	23	F	Segments 2–3^c^	IRA	10	3	1	1

a Complete response after 10 months treatment with sulindac.

b Studied after 4 months treatment with sulindac.

c Assessed with protein truncation test, mutation between codons 1099 and 1217 (overlapping part of segments 2 and 3 of APC protein). Listed are age, sex, mutational status, surgical status, polyp count before and after 6 months sulindac and numbers of polyps studied.

F=female; IRA=ileorectal anastomosis; APC=Adenomatous polyposis coli.

. In all, 21 polyps from five patients were studied.

### Immunohistochemistry for *β*-catenin

Immunohistochemistry was performed on 5 *μ*m sections of formalin-fixed, paraffin-embedded samples as described previously (Entius *et al*, 2000). For antigen retrieval, the slides were boiled for 10 min in citrate buffer followed by an overnight incubation at 4°C with a primary monoclonal antibody against *β*-catenin, clone 14 (Transduction Laboratories, Lexington, KY, USA). The staining pattern in adjacent normal mucosa was used as an indication for specificity.

Immunostained slides were scored semiquantitatively using a scale from 0 to 3 (0: no expression; 1: <5% positive nuclei; 2: 5–25% positive nuclei; 3: >25% positive nuclei). Also, membranous staining was scored separately as normal or decreased in comparison to adjacent mucosa. Slides were assessed in a coded manner by two independent observers (JJK and GJAO) and discrepancies were solved by consensus. Comparisons of staining patterns between adenomas collected at baseline and during treatment with sulindac were made using the nonparametric Mann–Whitney test and Fisher's exact test. A *P*<0.05 was considered statistically significant; *P*-values were two-sided.

### Cell culture and TCF reporter analysis

The human CRC cell lines DLD1 and SW480 were grown in RPMI medium supplemented with 10% fetal calf serum, 100 U ml^−1^ penicillin and 100 *μ*g ml^−1^ streptomycin (all from Life Technologies, Paisley, UK). Colorectal carcinoma cells were transiently transfected with either 5 *μ*g pTOPflash or pFOPflash reporter plasmids (Upstate Biotechnology, Lake Placid, NY, USA) using lipofectamine (Invitrogen, Paisley, UK). To correct for differences in transfection efficiency, cells were cotransfected with a reporter plasmid containing a GFP gene. Transfection efficiency was determined in live cells by counting, with a fluorescence microscope fitted with phase-contrast optics, the number of GFP-positive cells as well as the total cell number in 10 independent optical fields. The resulting transfection efficiency was used to correct for differences in transfection in each culture well in each experiment. All experiments were performed in triplicate. Cells were grown with or without the addition of sulindac sulphide (100 *μ*M) for 24 or 48 h. In addition, cells were grown with different concentrations of sulindac sulphide (0–100 *μ*M) for 24 h. Sulindac is *in vivo* metabolised into sulindac sulphide and sulindac sulphone by the intestinal flora. As these bacteria are not present in *in vitro* experiments, sulindac cannot be converted into its active metabolite. Therefore, we used the active metabolite of sulindac, sulindac sulphide, in our *in vitro* experiments. The concentration range of sulindac sulphide in this study was equivalent to that used by others (Zhang *et al*, 1999; Smith *et al*, 2000; Hawcroft *et al*, 2002). Sulindac sulphide was prepared as 1000 × stock solution in dimethyl sulphoxide (DMSO). Control cultures contained DMSO at an equivalent dilution, resulting in a final DMSO concentration of 0.1%. After the indicated period of time, cells were counted and equal numbers of cells were lysed in luciferase reporter lysis buffer (Promega, Madison, NY, USA). Cell lysates were monitored for luciferase activity using luciferase assay substrate buffer (Promega, Madison, NY, USA). Light units were recorded in a luminometer.

### Western blot analysis

The CRC cells DLD1 and SW480 were grown with different concentrations of sulindac sulphide (0–100 *μ*M) for 24 h. In addition, cells were grown with or without 100 *μ*M sulindac sulphide for 24 or 48 h. Protein extracts were prepared by resuspending the cells (0.5 × 10^6^ cells grown to subconfluence in a T25flask) into lysis buffer (10 mM Tris pH 8.0; 15 mM NaCl; 1% NP40, 10% glycerol; 0.4 mg ml^−1^ sodium orthovanadate). Protein extracts (50 *μ*g) were separated by SDS–PAGE and blotted onto immobilon-P transfer membranes (Millipore corp., Bedford, USA) by tank blotting. Membranes were blocked in Tris-buffered saline (100 mM Tris-HCl pH 7.5; 150 mM NaCl) containing 0.1% Tween (Sigma, St Louis, MO, USA) and 5% nonfat dry milk, probed with monoclonal antibodies; 8E4 (against nonphosphorylated *β*-catenin, dilution 1 : 1000) (Alexis Biochemicals, San Diego, CA, USA) and AC-15 (against *β*-actin, dilution 1 : 3000) (Sigma), or polyclonal antibodies: H-102 (against *β*-catenin, dilution 1 : 1000), C12 (against Met, dilution 1 : 1000), M-20 (against cyclin D1, dilution 1 : 1000) (all Santa Cruz Biotechnology, Santa Cruz, CA, USA). Proteins were detected with horseradish peroxidase-conjugated secondary antibodies (Dakopatts, Glostrup, Denmark) and a standard chemiluminescence Western blotting protocol (ECL Western blotting, Amersham Pharmacia Biotech Inc., Aylesbury, UK). Blots were analysed by densitometry. Intensities were quantified using NIH Image software (NIH, USA). All signals were normalised for loading in comparison with the appropriate *β*-actin signal.

## RESULTS

### β-Catenin expression in adenomas before and after sulindac treatment

In all, 21 adenomas from FAP patients removed before (*n*=9) and after (*n*=12) treatment with sulindac for up to 6 months were evaluated. The median treatment duration was 3 months with a range of 2–6 months and a mean of 3.7 months. All adenomas were tubular or tubulovillous lesions, <1 cm, with mild to moderate dysplasia. No morphological differences were noted between adenomas removed before and after treatment. The nuclear accumulation of *β*-catenin was assessed semiquantitatively on immunostained slides. Immunostained slides were scored semiquantitatively as described in the Materials and methods section and summarised in Table 2

**Table 2:** Nuclear *β*-catenin expression in adenomas before and after sulindac treatment

	**Score**			
**No. of adenomas**	**0**	**1**	**2**	**3**
Before treatment	0	4	1	4
After treatment*	4	6	2	0

* *P*=0.018.

**Figure 1:**
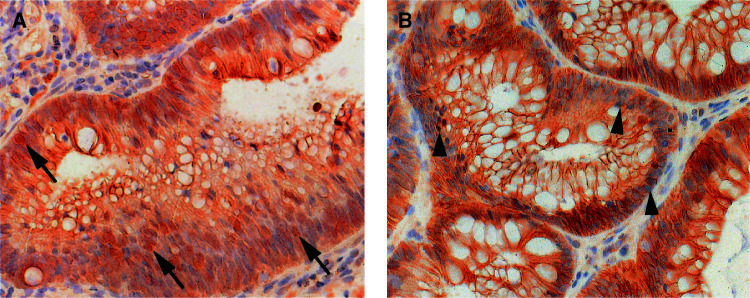
*β*-catenin expression in adenomas of FAP patients. Adenomas of FAP patients before (**A**) and after treatment with sulindac (**B**) were stained with an antibody against *β*-catenin. After sulindac treatment, the nuclear *β*-catenin staining (arrow) is strongly diminished (arrowhead).

. There was significantly more nuclear accumulation of *β*-catenin in baseline adenomas compared to adenomas collected after treatment with sulindac (*P*<0.02)(Figure 1

). Membranous *β*-catenin was decreased in nine out of nine baseline adenomas, and in nine out of 12 adenomas removed after sulindac (*P*>0.05). In the adjacent mucosa, a predominantly membranous staining without the nuclear accumulation of *β*-catenin was always observed.

### Sulindac targets *β*-catenin/TCF-activated transcription

**Figure 2:**
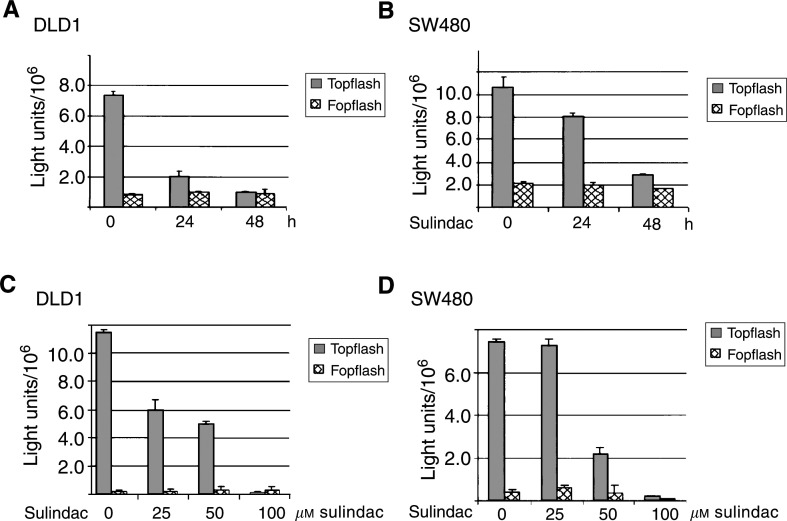
TCF reporter activity following sulindac treatment of DLD1 and SW480 cells. DLD1 (**A**) or SW480 cells (**B**) were treated with 100 *μ*M sulindac sulphide for 24 or 48 h. In parallel, DLD1 (**C**) or SW480 (**D**) cells were treated with various concentrations of sulindac sulphide (0–100 *μ*M) for 24 h. Cells were lysed and TOPflash and FOPflash activities were recorded in a luminometer.

The molecular mechanism of chemopreventive action of sulindac sulphide was analysed in human colorectal cancer cell lines DLD1 and SW480. These cell lines contain a constitutively activated Wnt-signalling pathway caused by mutations in the *APC* gene and are devoid of COX-2 expression (Boon *et al*, 2002; and confirmed by Western blotting, data not shown). The inhibition of *β*-catenin/TCF transcriptional activation by sulindac sulphide was monitored by transfecting a TCF reporter (pTOPflash) or, as a control, a construct containing scrambled TCF binding sites (pFOPflash) in both CRC cell lines. Differences in transfection efficiency were corrected for by cotransfecting the cells with a GFP-encoding reporter plasmid, as described in the Materials and methods section. Following sulindac sulphide (100 *μ*M) treatment for 24 or 48 h, *β*-catenin/TCF-activated TOPflash activity was abrogated in both DLD1 and SW480 cells (Figure 2A and B

). Comparable results were observed by indomethacin treatment of the CRC cell lines (data not shown). The inhibition of TCF-mediated transcription was also seen after treatment of both cell lines with different concentrations (0–100 *μ*M) of sulindac sulphide (Figure 2C and D). The effect of sulindac sulphide treatment on *β*-catenin/TCF-activated TOPflash activity was already visible after treatment of the cells with 25 *μ*M sulindac sulphide. FOPflash activity remained unchanged in both cell lines, indicating that sulindac sulphide treatment inhibited the Wnt-signalling pathway.

**Figure 3:**
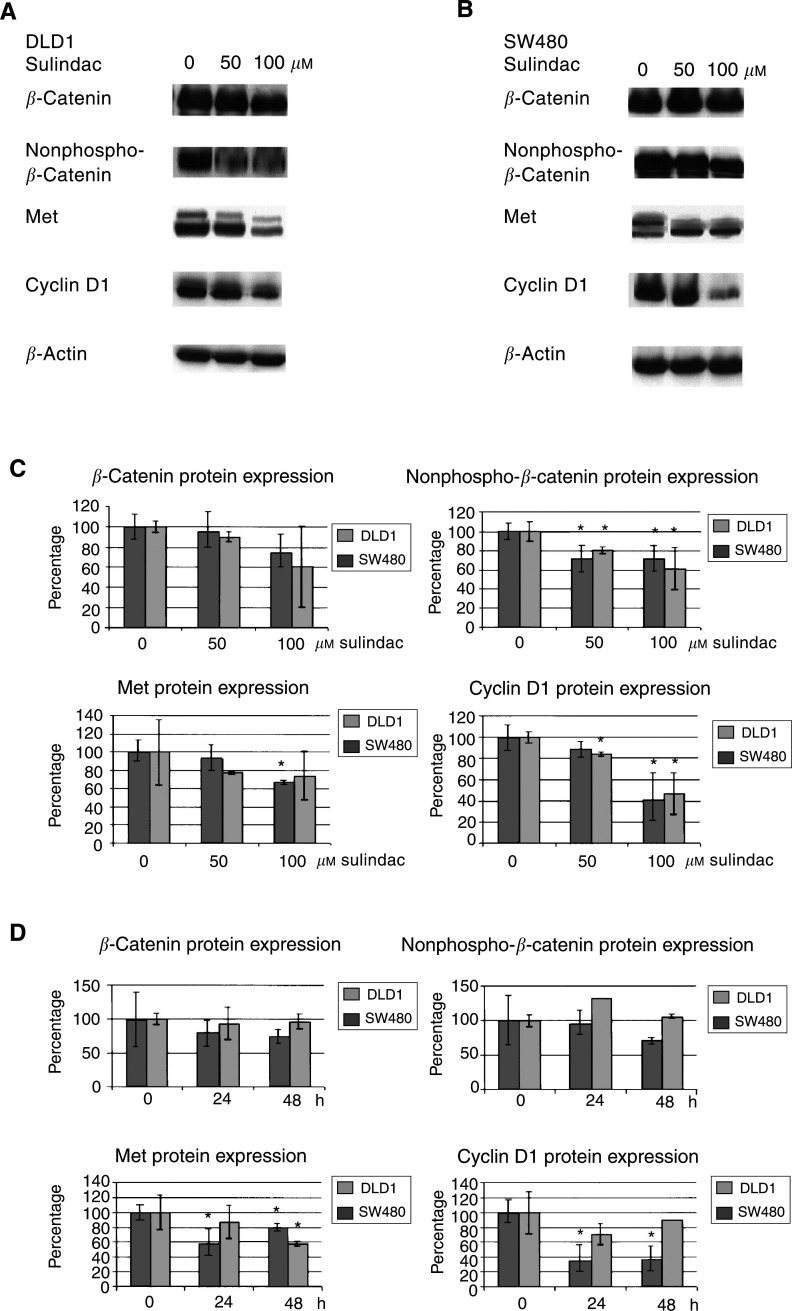
Kinetics of the effect of sulindac on TCF-regulated target genes. DLD1 (**A**) or SW480 cells (**B**) were treated with various concentrations of sulindac sulphide (0–100 *μ*M) for 24 h. Protein lysates were prepared and Western blot analyses performed with antibodies recognising total *β*-catenin, nonphosphorylated *β*-catenin, Met and cyclin D1. Blots were analysed by densitometry and each signal was normalised for loading in comparison with the appropriate *β*-actin signal (**C**). Data shown are mean values±s.e.m. from three independent experiments. In parallel, DLD1 or SW480 cells were treated with sulindac sulphide (100 *μ*M) for 24 or 48 h (**D**).

To further support this conclusion, we determined the effect of sulindac sulphide treatment on (i) the phosphorylation status of *β*-catenin and on (ii) the expression of TCF target genes. In the absence of Wnt signalling, *β*-catenin is phosphorylated by glycogen synthase kinase-3*β* (GSK-3*β*) and targeted for degradation by the ubiquitin–proteasomal pathway. Mutations in the *APC* gene, present in both DLD1 and SW480 cells, lead to a reduced phosphorylation of *β*-catenin and to the accumulation of nonphosphorylated *β*-catenin in the nucleus. Following sulindac sulphide treatment, a decrease in nonphosphorylated *β*-catenin expression in both cell lines was noted (Figure 3

). Furthermore, the expression levels of the TCF target genes Met (Boon *et al*, 2002) and cyclin D1 (Shtutman *et al*, 1999) were also downregulated in a time- and dose-dependent manner (Figure 3). A comparable dose- and time-dependent decrease in nonphosphorylated *β*-catenin and in the expression levels of the TCF target genes Met and cyclin D1 was also noted in response to indomethacin treatment (data not shown).

## DISCUSSION

In addition to COX-2, a number of alternative targets have been implicated in the chemopreventive action of NSAIDs, including Bcl-2, NF-*κ*B, NAG-1 and PPAR*δ* (Kopp and Ghosh, 1994; He *et al*, 1999; McEntee *et al*, 1999; Baek *et al*, 2001), suggesting that various distinct molecular pathways may play a role in the antitumour effects of these drugs. The present study provides *in vivo* and *in vitro* evidence that the *β*-catenin/TCF-4-signalling pathway is a target of sulindac.

We observed that the nuclear *β*-catenin expression in adenomas of FAP patients treated with sulindac is strongly decreased in comparison to the *β*-catenin expression in pretreatment adenomas of the same patients. A similar decrease in nuclear *β*-catenin has also been reported in rodent intestinal tumours (Brown *et al*, 2001). Previously, we found less nuclear *β*-catenin in sulindac-resistant adenomas compared to baseline adenomas (Keller *et al*, 2001). Those sulindac-resistant adenomas were collected during treatment with sulindac for up to 4 years, from selected sulindac-resistant patients, with a median treatment duration of 20 months, a range of 3–49 months and a mean of 26 months. Therefore, our previous study could not distinguish whether decreased nuclear *β*-catenin was caused by sulindac or reflected intrinsic features of sulindac-resistant adenomas. The present study compared adenomas from FAP patients removed before and after treatment with sulindac for up to 6 months. At that time point, the maximum efficacy of sulindac on the number and size of adenomas was noted (Giardiello *et al*, 1993), and in general resistance started to develop afterwards. Thus, it is assumed that the observed decrease of nuclear *β*-catenin in the present investigation is mostly accounted for by the sulindac treatment, instead of being related to resistance. This corresponds to data from McEntee *et al* (1999) showing decreased nuclear and cytoplasmic *β*-catenin in adenomas of *Apc*^*Min*^ mice upon treatment with sulindac for several days.

The observation that sulindac treatment leads to a decrease in nuclear *β*-catenin *in vivo* is of great interest, since it suggests a direct link between the tumour suppressive effects of sulindac and the key defect of colorectal cancer, that is, deregulated Wnt signalling. Mutations involving components of the Wnt-signalling cascade, specifically in APC or *β*-catenin, are essential for the initiation of colorectal cancer. In the normal intestinal epithelium, these molecules are part of a multiprotein complex. In this complex, *β*-catenin is phosphorylated by GSK-3*β* and targeted for degradation by the ubiquitin–proteasomal pathway. Mutations in APC or *β*-catenin lead to dissociation of the complex, causing the accumulation of nonphosphorylated *β*-catenin, which translocates to the nucleus and acts as a transcriptional coactivator of TCF transcription factors (Kinzler and Vogelstein, 1996; Bienz and Clevers, 2000). Our observation that sulindac treatment diminishes the nuclear accumulation of the transcriptional coactivator *β*-catenin in adenomas of FAP patients *in vivo* strongly suggests that NSAIDs exert tumour suppressive effects by interfering with TCF-mediated transcription. In line with these *in vivo* data, we observed that sulindac sulphide treatment of the CRC cell lines DLD1 and SW480 strongly suppresses TCF reporter activity. Furthermore, NSAID treatment decreased the expression of the TCF target genes Met and cyclin D1 in a time- and dose-dependent manner. These suppressive effects were not due to a generalised transcriptional repression, since FOPflash reporter activities remained unchanged after sulindac sulphide treatment in the CRC cells. The effect of NSAIDs is less pronounced on the TCF-target protein levels than on TCF-regulated transcription, as shown in the TCF reporter assay. This effect can be explained by slow protein turnover rates.

Sulindac sulphide treatment led to only a moderate decrease in total *β*-catenin levels. This finding was recently also reported by Smith *et al* (2000), although Dihlmann *et al* (2001) reported no appreciable effect on *β*-catenin levels, despite the change in TCF-mediated transcription. Although we have no explanation for this discrepancy, the validity of our finding is strongly supported by the observation that the decrease in nonphosphorylated *β*-catenin (which represents the transcriptionally active portion of *β*-catenin) was more pronounced than that of the total *β*-catenin pool, suggesting a selective effect of sulindac sulphide on the pool of *β*-catenin that is involved in Wnt signalling. Since the CRC cells used in this study carry an *APC* mutation, preventing the formation of the APC/*β*-catenin multiprotein complex, it seems unlikely that the NSAID-induced decrease in nonphosphorylated *β*-catenin is regulated by GSK-3*β* activity. Other pathways of *β*-catenin downregulation might involve caspase-mediated cleavage (Schmeiser *et al*, 1998) or the inhibition of guanosine 3′,5′-cyclic monophosphate (cGMP) phosphodiesterase, leading to increased cGMP levels and downregulation of *β*-catenin, possibly via protein kinase G phosphorylation (Thompson *et al*, 2000). Also, NSAIDs may directly inhibit the translocation of *β*-catenin to the nucleus.

Taken together, our findings demonstrate that sulindac suppresses TCF-mediated transcription, presumably by preventing the nuclear accumulation of active *β*-catenin. Our observations provide new insights in the pathways involved in chemoprevention of NSAIDs and could advance the design of better chemopreventive drugs.
